# *Streptococcus suis*, an Important Cause of Adult Bacterial Meningitis in Northern Vietnam

**DOI:** 10.1371/journal.pone.0005973

**Published:** 2009-06-22

**Authors:** Heiman F. L. Wertheim, Huyen Nguyen Nguyen, Walter Taylor, Trinh Thi Minh Lien, Hoa Thi Ngo, Thai Quoc Nguyen, Bich Ngoc Thi Nguyen, Ha Hong Nguyen, Ha Minh Nguyen, Cap Trung Nguyen, Trinh Tuyet Dao, Trung Vu Nguyen, Annette Fox, Jeremy Farrar, Constance Schultsz, Hien Duc Nguyen, Kinh Van Nguyen, Peter Horby

**Affiliations:** 1 Oxford University Clinical Research Unit, National Institute of Infectious and Tropical Diseases, Hanoi, Viet Nam; 2 Centre for Tropical Medicine, Nuffield Department of Clinical Medicine, Oxford University, Oxford, United Kingdom; 3 National Institute of Infectious and Tropical Diseases, Hanoi, Vietnam; 4 Oxford University Clinical Research Unit, Hospital of Tropical Diseases, Ho Chi Minh City, Viet Nam; 5 Centre for Poverty-Related Communicable Diseases, Academic Medical Center, Amsterdam, The Netherlands; Columbia University, United States of America

## Abstract

**Background:**

*Streptococcus suis* can cause severe systemic infection in adults exposed to infected pigs or after consumption of undercooked pig products. *S. suis* is often misdiagnosed, due to lack of awareness and improper testing. Here we report the first fifty cases diagnosed with *S. suis* infection in northern Viet Nam.

**Methodology/Principal Findings:**

In 2007, diagnostics for *S. suis* were set up at a national hospital in Hanoi. That year there were 43 *S. suis* positive cerebrospinal fluid samples, of which *S. suis* could be cultured in 32 cases and 11 cases were only positive by PCR. Seven patients were blood culture positive for *S. suis* but CSF culture and PCR negative; making a total of 50 patients with laboratory confirmed *S. suis* infection in 2007. The number of *S. suis* cases peaked during the warmer months.

**Conclusions/Significance:**

*S. suis* was commonly diagnosed as a cause of bacterial meningitis in adults in northern Viet Nam. In countries where there is intense and widespread exposure of humans to pigs, *S. suis* can be an important human pathogen.

## Introduction

*Streptococcus suis* infection is a zoonosis which can cause severe systemic infection in humans exposed to infected pig tissue [1]. To date there have been relatively few reports of *S. suis* infection in humans, with around 700 cases reported worldwide, most of them in the last few years [1], [2], [3], [4]. In developed countries most cases are described in people with occupational exposure to pigs, such as pig farmers and abattoir workers. One study estimated the annual risk of developing *S. suis* infection in abattoir workers and pig farmers in a developed country to be approximately 3/100.000 per year [5].

In developing countries with intense pig farming, like those in Southeast Asia, the risk of acquiring *S. suis* infection is unknown as it is not a notifiable disease and under diagnosis is common. However the two largest published case series are from this region and together account for more than 50% of all reported cases [1], [3]. It is therefore possible that *S. suis* infection is a considerable, unrecognized burden in large parts of Southeast Asia [1], [2]. Whilst a recent study in southern Viet Nam showed that *S. suis* was the most common cause of bacterial meningitis in adults, *S. suis* has never been reported in northern Viet Nam [2]. Northern Viet Nam is more than 1000 miles from southern Viet Nam and experiences a very different climate, which may influence the local epidemiology of bacterial meningitis. In order to determine whether *S. suis* is a common cause of bacterial meningitis in northern Viet Nam we established enhanced diagnostics for *S. suis* at the National Institute of Infectious and Tropical Disease (NIIITD), a tertiary referral hospital in Hanoi. Here we report the data from 2007.

## Methods

This study was conducted at the National Institute of Infectious and Tropical Diseases (NIITD), Hanoi, from January 2007 to December 2007. The NIITD is a 160 bed tertiary care center for adult patients with infectious diseases and also serves as a referral center for central nervous system infections in northern Viet Nam. Admitted patients with suspected meningitis were managed by local physicians, according to local practice that included the taking of cerebrospinal fluid (CSF) by lumbar puncture. Patient data were collected retrospectively using a pre-printed data collection form from their medical records. For sepsis classification we used standard criteria [6].

Specimens were processed using standard microbiological methods. Optochin negative alpha-haemolytic streptococci on blood agars, isolated from blood and CSF were tested with API 20 Strep® [Biomerieux, France] for identification at the NIITD laboratory. *S. suis* isolates were tested for penicillin and ceftriaxone susceptibility by E-test (AB Biodisk, Solna, Sweden) on Mueller Hinton agars (Biorad, USA). Real time polymerase chain reaction (PCR) diagnostics for *S. suis* serotype 2 with *cps2J* as gene target (primers and probe: *forward GGTTACTTGCTACTTTTGATGGAAATT*, *reverse* *CGCACCTCTTTTATCTCTTCCAA*, probe *FAM-TCAAGAATCTGAGCTGCAAAAGTGTCAAATTGA-TAMRA*) were implemented according to a previously described method [2]. Furthermore, isolated *S. suis* strains were genotyped by pulsed field gel electrophoresis (PFGE), after *SmaI* digestion. Four representative strains from the main clusters in southern Viet Nam (A, B, C, and D) were included [2]. Also reference strains were included (89–151 and 31533). A dendrogram was generated by Dice analysis (optimization, 0.5%; band tolerance, 1.5%) and cluster analysis with unweighted pair group method with arithmetic mean, using Bionumerics software (Applied Maths).

*S. suis* cases were geo-coded using the patient's address and overlaid onto a map depicting the estimated number of pigs per square kilometer (pig density data from the National Statistics Office, Hanoi, Viet Nam). Statistical differences in proportions were assessed by Fisher exact test. P values below 0.05 were considered significant. Institutional Review Board approval for this study was obtained.

### Ethical considerations

This study was approved by the Oxford Tropical Research Ethics Committee and the Scientific Committee of the National Institute of Infectious and Tropical Diseases. Written informed consent was obtained.

## Results

**Figure 1:**
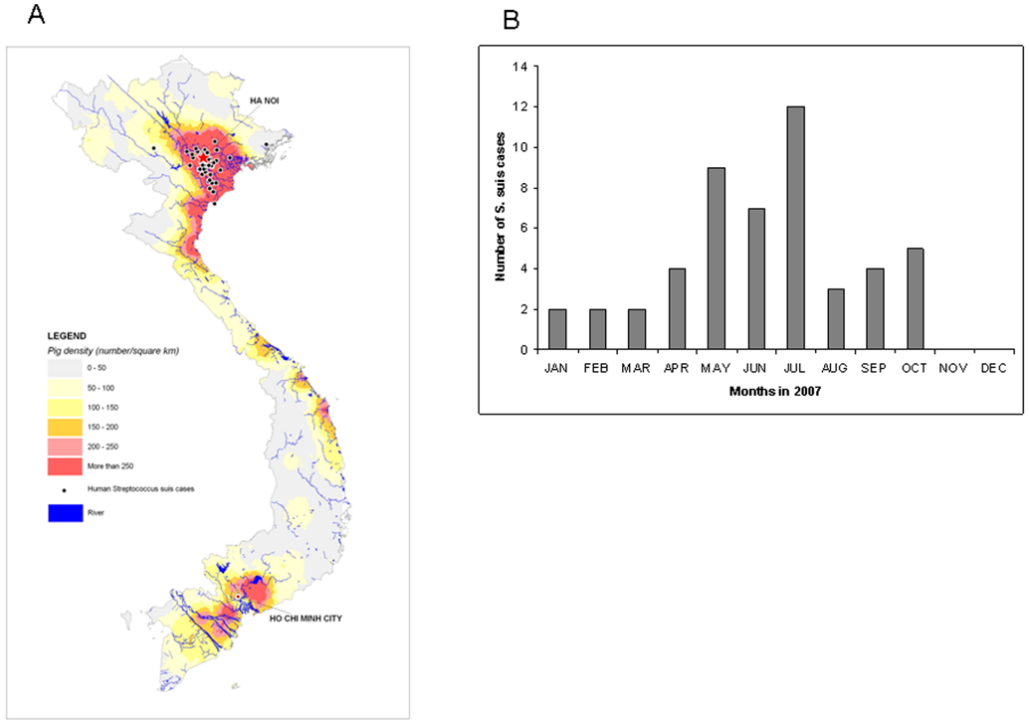
Location and seasonality of S. suis infections. (A) Location of residence of 50 human S. suis cases diagnosed in northern Viet Nam in 2007 (black dot is one case) and pig density; (B) Month of diagnosis of S. suis cases during 2007.

Between January and December 2007, 562 CSF specimens were submitted to the microbiology laboratory for analysis. Eleven specimens were positive for *Cryptococcus neoformans* (1.9%), three for *Streptococcus pneumoniae* (0.5%), three for *Streptococcus species* (0.5%), one for *Enterobacter cloace* (0.2%) and 43 for *S. suis* (7.7%). Of the 43 *S. suis* positive CSF samples, *S. suis* was isolated in 32 and 11 were only positive by PCR. An additional seven patients were blood culture positive for *S. suis* but CSF culture and PCR negative; making a total of 50 (8.9%) patients with laboratory confirmed *S. suis* infection (Table 1). The number of *S. suis* cases peaked during the summer months of May to July (Figure 1B). The 50 patients with *S. suis* disease were mostly older males (44 males [88.0%], median age all patients: 48 years, range: 17–78 years). The majority was farmer (n = 35, 70.0%) and a total of 16 patients reported a recent exposure to pigs or pork (32.0%): eight male patients had slaughtered pigs, five patients were exposed to pork products, and three consumed raw pig blood. Thirteen patients (26%) reported excessive alcohol consumption. None of the patients had a history of splenectomy.

**Table 1:** Diagnosis of 50 S. suis cases in Hanoi in 2007.

Diagnosis by:	Number of *S. suis* patients
Blood culture only	7
Cerebrospinal fluid culture*	32
Cerebrospinal fluid PCR only	11

* Positive cerebrospinal fluid cases were also regularly positive by blood culture (data not shown).

Most patients presented with fever and meningism (Table 2). Complications developed in 15 (30%) patients, including renal and liver impairment (n = 14 and n = 7, respectively), septic shock (n = 6), ARDS (n = 3), endophthalmitis (n = 2) and a spinal abscess (n = 1). Seven patients (14%) had a purpuric/ecchymotic skin rash or purpura fulminans. Eight (16%) patients experienced respiratory failure requiring mechanical ventilation. Twenty-six patients (52%) recovered completely, 21 patients (42%) recovered with sequelae, and three patients died with septicemia. Hearing loss was the most common sequela (n = 19, 38%); others were: paralysis (n = 2), loss of vision (n = 2), dysarthria with gait ataxia (n = 2), abscess spinal column (n = 1), and residual renal impairment (n = 1). The most common empirical antibiotic treatment was ceftriaxone in combination with ampicillin. The latter antibiotic was given empirically to cover for Listeriosis. Twenty-six (52%) of the patients also received corticosteroids, as determined by the attending physician based on disease severity. Hearing loss occurred both in patients treated and not treated with corticosteroids, 11/26 (42.3%) versus 8/24 (33.3%).

**Table 2:** Characteristics of 50 patients with S. suis infection.

General	Frequency	
Male sex	44	(88%)
Age in years – mean (range)	48	(17–78)
Farmer	35	(70%)
Pig contact	16	(32%)
**Clinical features on admission**		
Duration of symptoms - median	4 d	(1–17)
Fever	50	(100%)
Temperature at admission (°C)	38.3	(36.5–40)
Heart rate (beats/min)	100	(78–120)
Neck stiffness	44	(88%)
Kernig's sign	42	(84%)
Headache	46	(92%)
Confusion	23	(46%)
Glasgow Coma Scale score	13.5	(6–15)
Skin rash	7	(14%)
Septic shock∧	6	(12%)
**Laboratory indices on admission - mean (range)**		
*Blood*		
CRP	75	(6–192)
CPK	696.1	(46–2318)
AST (U/L)	128.5	(17–1122)
ALT (U/L)	66.9	(11.6–247)
Leukocyte count (×10^9^/L)	17.9	(8.3–44.4)
Platelet count (×10^6^/L)	169.8	(4–770)
*CSF*		
Leukocyte count (×10^9^/L)	3.2	(0.4–2.7)
Protein (g/L)	1.7	(0.6–5.1)
Glucose (mmol/L)	1.7	(0.2–4.7)
**Outcome**		
Duration of admission	17 d	(2–39)
Complete recovery	26	(52%)
Recovery with sequelae*	21	(42%)
Died	3	(6%)

∧ Sepsis category defined according to standard criteria [6].

* Mostly hearing loss (n = 16).

**Figure 2:**
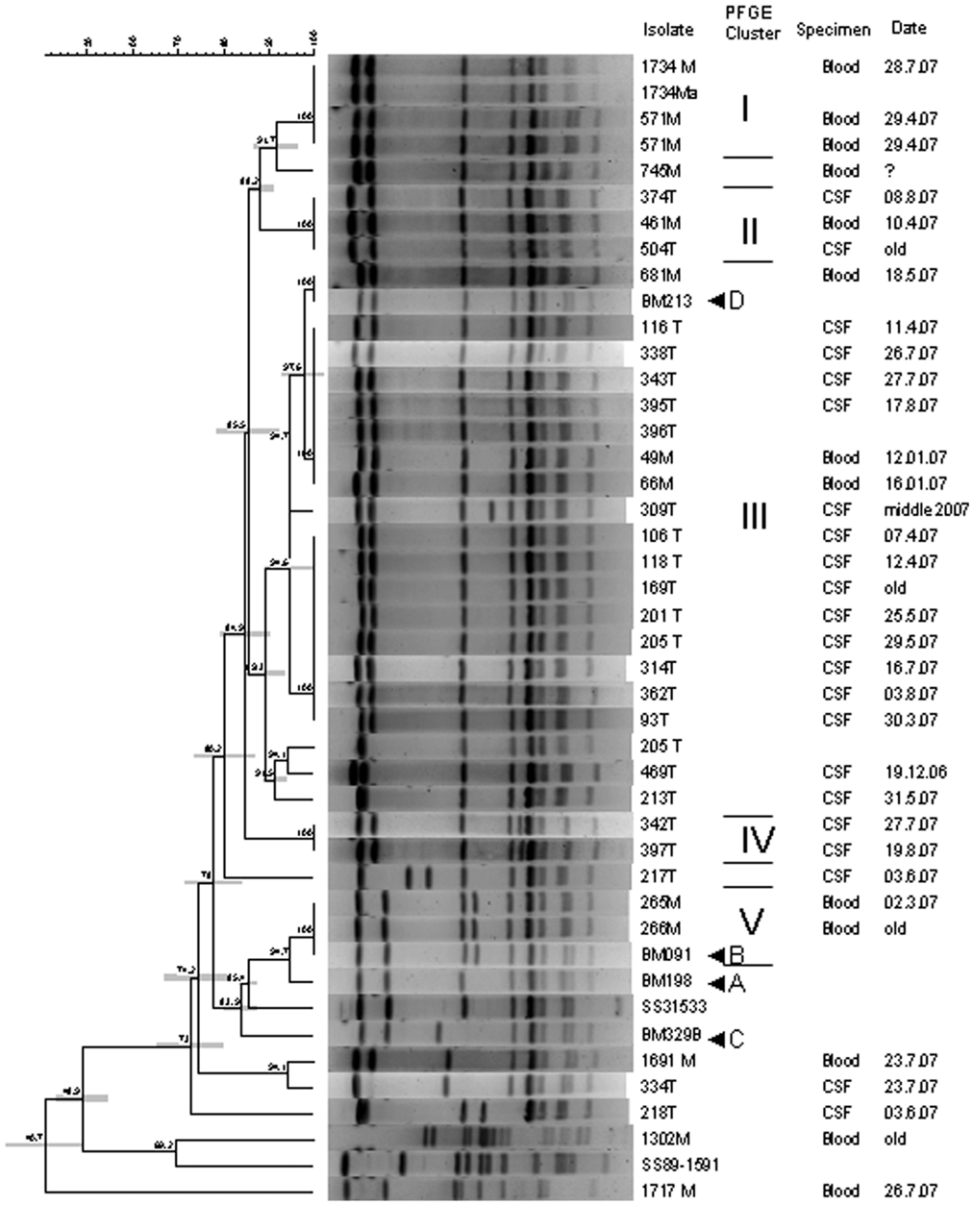
PFGE after SmaI digestion of Streptococcus suis serotype 2 strains isolated from patients in northern Vietnam. A dendrogram was generated by Dice analysis (optimization, 0.5%; band tolerance, 1.5%) and cluster analysis with unweighted pair group method with arithmetic mean, using Bionumerics software (Applied Maths). Bars indicate 95% CIs. Four representative strains from the main clusters in southern Viet Nam (A, B, C, and D) were included. Also reference strains were included (89–151 and 31533). Strains with the annotation old are from before 2007.

Laboratory findings at admission showed leukocytosis in 40 patients (80%) and thrombocytopenia in 29 patients (58%, Table 2). The CSF in patients showed high protein concentration (mean 1.7 g/l, range 0.6–5.1), low glucose levels (mean 1.7 mmol/l, range 0.2–4.7) and neutrophilia (mean white blood cell count 3253/mm^3^, range 0.4–26,500). Microscopic examination of Gram- stained CSF specimens revealed Gram-positive cocci in 28 patients (56.0%). Tested *S. suis* isolates were susceptible to penicillin and ceftriaxone (data not shown). PFGE analysis demonstrated that the bacterial population structure in northern Viet Nam has important similarities to that seen in the south (Figure 2). There was one large cluster III, similar to the dominant group D seen in southern Vietnam[2]. The group A cluster that was seen in southern Vietnam was not found in the north. Most patients lived in the Red River delta, where there is a high density of pigs (Figure 1). There was no obvious spatial or temporal clustering of cases to suggest an outbreak.

## Discussion

Data from our study complement those of an earlier report from a tertiary referral hospital in southern Viet Nam and demonstrate that *S. suis* type 2 is the most commonly detected organism in acute adult bacterial meningitis in both north and south Vietnam (unpublished data). Together they represent a case series of 193 patients and illustrate that *S. suis* meningitis is an endemic zoonosis of adults in Vietnam, which may also be true for other Southeast Asian countries where pig farming is common. This information has important public health consequences since *S. suis* infection is preventable through applying protective measures during the slaughtering and processing of pigs, and through proper cooking of pig meat and other pig body parts.

There are estimated to be around 26.8 million pigs in Vietnam, of which 40% live in the Red River and Mekong River Delta (source: FAOSTAT 2006). Many rural households have a small number of pigs, thus putting a high proportion of the Vietnamese population at risk. In our study, there were considerably more patients during the summer months from May to July than during the rest of the year. Whether this pattern observed in 2007 is typical of other years and represents seasonality in risk of infection remains to be determined.

During the study period there was a Porcine Respiratory and Reproductive System (PRRS) virus outbreak in the pig population in the same region [7]. This respiratory syndrome is often associated with severe secondary infection with bacterial agents like *S. suis* [8]. Therefore the PRRS virus outbreak may have led to an increased the risk of transmission of *S. suis* to humans through exposure to pigs with PRRS virus infection and concomitant *S. suis* disease. This hypothesis requires further investigation.

The large majority of *S. suis* cases were male and this probably represents gender-associated behavioural or occupational risk factors. The finding of a history of excessive alcohol consumption in 26% of patients may indicate that alcoholism is a risk factor for *S. suis* infection or disease. Almost 70% of cases did not report recent contact with pigs or pork products and therefore further work is needed to better define the risk factors for acquiring *S. suis* infection, like consumption of uncooked pig meat or blood.

*S. suis* can be cultured easily from cerebral spinal fluid (CSF) or blood with standard microbiological techniques. *S. suis* grows on blood agar as small, greyish and mucoid colonies with a zone of alpha-haemolysis and is optochin resistant. Determination to the species level is performed with biochemical tests, like optochin, Voges-Proskauer, salicin, trehalose, and 6.5% NaCl. Commercial systems, like API Strep® (Biomerieux, France), can also be used. These tests, including simple biochemical reactions for presumptive identification, are usually not available in developing countries and *S. suis* may therefore remain often undiagnosed or misdiagnosed. Furthermore, false negative culture results may occur due to antibiotic use prior to obtaining the specimens for culture. Approximately twenty percent of the cases reported here would have been missed without access to PCR, illustrating the important diagnostic potential of this technique. Fortunately, *S. suis* is generally susceptible to the readily available antibiotics penicillin and ceftriaxone. However, the severity of the sepsis syndrome seen in some patients requires careful clinical management and three patients in our series died despite being managed in an intensive care unit.

Most of our patients presented with severe disease and typical symptoms of meningitis. The mortality rate in our case series was 6%; higher than the reported 2.6% mortality in south Viet Nam. Hearing loss was the most common sequelae at discharge, affecting one third of the patients. The higher rates of hearing loss (66%) reported elsewhere probably reflect the use of audiometry to detect milder degrees of hearing loss than we were able to detect without access to an audiometer [2]. Our observation of a higher rate of hearing loss in the patients treated with corticosteroids cannot be interpreted since we have not been able to adjust for the severity of illness or hearing impairment prior to commencing corticosteriod treatment. In a recent, placebo controlled clinical trial, high dose dexamethasone significantly reduced the rate of post treatment hearing loss (12 vs. 38%) and therefore corticosteriod treatment should be used in all cases of *S. suis* meningitis [9].

In conclusion, *S. suis* is an important cause of adult meningitis in both north and south Viet Nam. Hearing loss as an early complaint is an important clue in the history in this setting. Laboratory capacity building to culture and identify *S. suis* from CSF and blood would aid greatly the ability to diagnose *S. suis* correctly and give reliable estimates of its burden in the community. This may also apply to other Southeast Asian countries. As far as we know the main reservoir for *S. suis* is pigs and it is not normally carried by humans. Countries where pig farming is common and *S. suis* has not yet been identified as a cause of bacterial meningitis should establish diagnostic assays to identify this bacterium. Identifying this disease is essential as it may be readily preventable through actions directed at the rearing and slaughter of pigs and food preparation practices.
